# Anticancer Activity of Pyrimethamine via Ubiquitin Mediated Degradation of AIMP2-DX2

**DOI:** 10.3390/molecules25122763

**Published:** 2020-06-15

**Authors:** Dae Gyu Kim, Chul Min Park, Srigouri Huddar, Semi Lim, Sunghoon Kim, Sunkyung Lee

**Affiliations:** 1 Medicinal Bioconvergence Research Center, College of Pharmacy and College of Medicine, Gangnam Severance Hospital, Yonsei University, Incheon 21983, Korea; kimeorb@hanmail.net (D.G.K.); smlim226@naver.com (S.L.); 2 Center for Convergent Emerging Virus Infection, Korea Research Institute of Chemical Technology, Daejeon 34114, Korea; parkcm@krict.re.kr; 3 Drug Information research Center, Korea Research Institute of Chemical Technology, Daejeon 34114, Korea; srigouri@krict.re.kr; 4 Korea University of Science and Technology, Daejeon 34114, Korea

**Keywords:** pyrimethamine, AIMP2-DX2, ubiquitination, anticancer, drug repositioning

## Abstract

While aminoacyl-tRNA synthetase-interacting multifunctional protein 2 (AIMP2) is a tumor suppressor, its exon 2-depleted splice variant (AIMP2-DX2 or shortly DX2) is highly expressed in human lung cancer, and the ratio of DX2 to AIMP2 increases according to the progression of lung cancer. In this study, pyrimethamine inhibited the level of DX2 (IC_50_ = 0.73 µM) in A549 cells expressing nanoluciferase-tagged DX2. In a panel of 5 lung cancer cell lines with various DX2 levels, pyrimethamine most potently suppressed the growth of H460 cells, which express high levels of DX2 (GI_50_ = 0.01 µM). An immunoblot assay in H460 cells showed that pyrimethamine decreased the DX2 level dose-dependently but did not affect the AIMP2 level. Further experiments confirmed that pyrimethamine resulted in ubiquitination-mediated DX2 degradation. In an in vivo mouse xenograft assay using H460 cells, intraperitoneal administration of pyrimethamine significantly reduced the tumor size and weight, comparable with the effects of taxol, without affecting body weight. Analysis of tumor tissue showed a considerably high concentration of pyrimethamine with a decreased levels of DX2. These results suggest that pyrimethamine, currently used as anti-parasite drug, could be repurposed to treat lung cancer patients expressing high level of DX2.

## 1. Introduction

Aminoacyl-tRNA synthetases (ARSs) are the enzymes that catalyze the ligation of specific amino acids to their cognate tRNAs in the first step of protein translation [1]. In higher eukaryotic systems, nine ARSs and three aminoacyl-tRNA synthetase-interacting multifunctional proteins (AIMPs) are assembled into a multi-tRNA synthetase complex (MSC) that serves as a hub for various regulatory pathways in addition to playing a role in protein synthesis. While three AIMPs, AIMP1/p43, AIMP2/p38 and AIMP3/p18, function as scaffold proteins for the assembly and integrity of MSCs [2,3,4], they also participate in diverse regulatory roles that are not directly linked to protein synthesis.

Among the AIMPs, AIMP2 works as a potent tumor suppressor and induces growth arrest by transforming growth factor-β (TGF β) signaling via ubiquitin-mediated degradation of FUSE-binding protein (FBP) and downregulation of c-myc [5]. In addition, AIMP2 suppresses tumor necrosis factor-α (TNF-α)-dependent cell proliferation via degradation of TNF receptor-associated factor 2 (TRAF2) [6]. AIMP2 also functions as a proapoptotic factor by protecting the tumor suppressor p53 under DNA damage stress [7]. AIMP2 heterozygous mice showed increased susceptibility to tumorigenesis driven by lung, colon, and skin carcinogens, showing that the antiproliferative and proapoptotic activities of AIMP2 are also evident in vivo [8].

While AIMP2 is a tumor suppressor, an exon 2-depleted splice variant of AIMP2 (AIMP2-DX2, hereafter abbreviated as DX2) is highly expressed in human lung cancer. Like AIMP2, DX2 can also bind to FBP, TRAF2, and p53, thereby compromising the tumor-suppressive interaction of AIMP2 with these proteins. The expression ratio of DX2 to AIMP2 increases with the degree of malignancy of lung and chemoresistant ovarian cancer [9,10]. Collectively, these data suggest that DX2 might be an effective anticancer therapeutic target. We recently reported that BC-DXI01, a synthetic compound, showed antitumorigenic activity in a DX2-driven tumor xenograft mouse model as well as DX2-specific inhibitory activity through decreasing the DX2 mRNA level [11]. Then, we attempted to identify a DX2 inhibitor for the treatment of lung cancer using clinical collections (a drug repositioning library) from Korea Chemical Bank (KCB) [12] at Korea Chemical Institute of Chemical Technology (KRICT, Daejeon, Korea). Through the improved primary screening system in A549 cells expressing luciferase-tagged DX2 described in our previous paper [11], we identified pyrimethamine as a hit. Pyrimethamine was launched in 1953 for the treatment of malaria as a dihydrofolate reductase (DHFR) inhibitor (www.drugbank.com), and more recently for the treatment of toxoplasmosis combined with a sulfonamide [13,14]. Pyrimethamine also has been widely studied for the treatment of advanced solid tumors such as lung cancer, ovarian cancer, triple negative breast cancer, and chronic lymphocytic leukemia based on the various mechanisms including signal transducer and activator of transcription 3 (STAT3) inhibition [15,16,17,18,19,20,21,22,23], differently from DX2 inhibition.

In this paper, we describe the effects of pyrimethamine in a nanoluciferase assay, a viability assay in 5 lung cancer cell lines with different expression levels of DX2, a mechanism of action study, and an in vivo xenograft assay.

## 2. Results

### 2.1. Identification of Pyrimethamine as a Suppressor of DX2 Level

**Figure 1:**
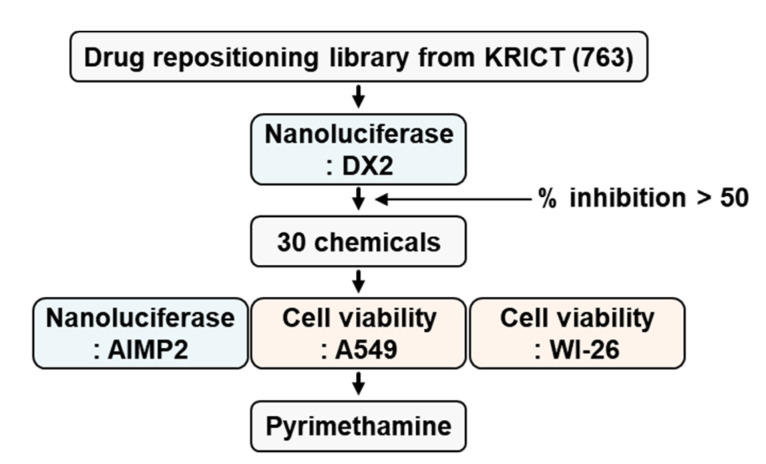
Screening to identify DX2 inhibitors. 763 chemicals (5 µM) from KCB at KRICT was used for first screening. Thirty chemicals showing greater than 50% inhibition were subjected to secondary screening via nanoluciferase and cell viability assays using A549 and WI-26 cells.

**Figure 2:**
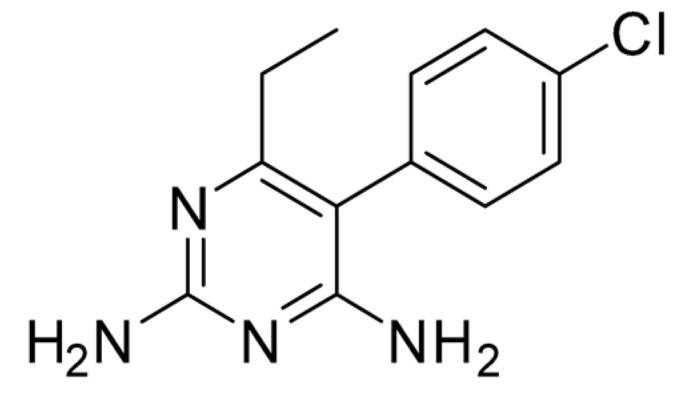
Structure of pyrimethamine.

Previously, we established a firefly luciferase assay system with A549 cells expressing luciferase-tagged DX2 to find small molecules that could reduce the cellular level of DX2 [11]. For establishment of more sensitive high-throughput screening, we changed the luciferase tag to a nanoluciferase tag because the nanoluciferase is three times smaller and 2,500,000 times brighter than the previously used luciferase, and 763 compounds were screened in the drug repositioning library from KCB at KRICT by our screening system. We found 30 compounds that reduced the cellular level of DX2 at 5 µM by more than 50% compared with the level in control cells (Figure 1). Pyrimethamine (Figure 2) showed a good suppressive effect on the level of DX2 (IC_50_ = 0.73 μM) but no inhibitory effect on the level of AIMP2 (IC_50_ > 100 µM), indicating that it selectively decreased the expression level of DX2.

### 2.2. Antiproliferative Effect of Pyrimethamine on Cancer Cells but not Normal Cells

A549 and WI-26 cells [9,24] were subjected to a cell viability assay to determine the GI_50_ values for 50% inhibition of cell growth. Pyrimethamine displayed good inhibition of cell growth in A549 cells (GI_50_ = 0.8 µM) similar to its IC_50_ (0.73 µM), which show a moderate level of DX2, but no inhibitory effect in WI-26 cells (GI_50_ > 100 μM), which have a low level of DX2 expression (Table 1). Additionally, cell viability experiments were performed using 4 other lung cancer cell lines (H460, HCC-1359, HCC-366, and H2087) with various levels of DX2 [9,24]. Pyrimethamine showed potent anti-proliferative activity on H460 cells with high levels of DX2 (GI_50_ = 0.01 μM), implying that the anti-proliferative potency of pyrimethamine is well correlated with the DX2 level, while taxol showed similar potencies on all 6 tested cell lines as (GI_50_ = 0.02~0.08 μM) shown in Table 1.

### 2.3. DX2 Level-Dependent Antiproliferative Effect of Pyrimethamine

**Figure 3:**
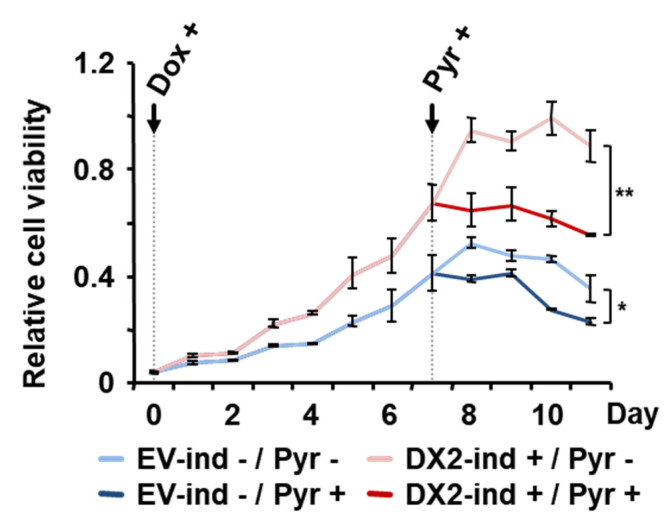
Significance of the DX2 level on pyrimethamine-mediated suppression of cancer cell viability. DX2- or EV-inducible (ind) A549 cells were treated with doxycycline (Dox) and pyrimethamine (Pyr) at the indicated time to induce the expression of DX2 and to reduce cell viability, respectively. The values on the graph were calculated against cell viability of empty vector (EV)-inducible cell lines at time 0 and presented as a relative cell viability. The experiments were independently repeated three times. Student’s two-tailed t-test was performed for statistical analysis (* *p* < 0.05, ** *p* < 0.01). The error bars denote the standard deviation (S.D.), the data shown are the mean ± S.D.

Based on the results above, the cell lines showing high level of DX2 appear to be highly susceptible for pyrimethamine-dependent cell death. To confirm whether anti-proliferative activity of pyrimethamine is mediated by DX2, we set up the cell proliferation assay using DX2-inducible system [24]. For negative control against the expressed plasmid, empty vector (EV) was used. DX2- or EV-inducible stable A549 cells were treated with doxycycline (Dox) to induce the expression of DX2 and the cells were subsequently treated with pyrimethamine. As expected, the DX2-induced A549 cell lines showed the increased cell viability. After treatment with pyrimethamine, we observed that pyrimethamine significantly inhibited the growth of DX2-inducible A549 cells compared to EV-inducible A549 cells (Figure 3). Pyrimethamine was reported to show the anti-tumor effect against cancer cells [15,16,17,18,19,20,21,22,23], but its mechanism was still veiled [19]. Our results showed that the increased level of DX2 leads the strong anti-tumor effect, suggesting that DX2 could be one of the targets for pyrimethamine-mediated anti-tumoral effect. This results indicated that anti-proliferative activity of pyrimethamine would depend on the expression level of DX2.

### 2.4. Specific Suppression of DX2 Level by Pyrimethamine

**Figure 4:**
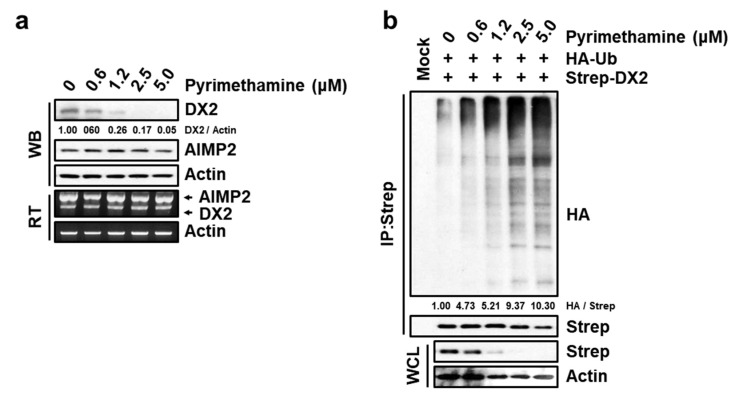
Specific inhibitory effect of pyrimethamine on DX2 protein expression. (**a**), Pyrimethamine-dependent level of DX2 protein and mRNA. Pyrimethamine-treated (12 h) H460 cells were subjected to Western blotting (WB) and Reverse transcriptase polymerase chain reaction (RT-PCR) for detecting the indicated proteins and mRNAs, respectively. Actin was used as the loading control. (**b**), Pyrimethamine-mediated ubiquitination of DX2. 293T cells expressing Strep-DX2 and HA-ubiquitin (Ub) were treated with pyrimethamine and MG-132 for 12 h. HA attached the ubiquitin as a tag is the abbreviated name from hemagglutinin. IP and WCL means immunoprecipitation and whole cell lysates, respectively. (**a**,**b**), The numbers below the image panel mean the quantified value.

To examine the concentration-dependent inhibitory effect of pyrimethamine on the protein and mRNA levels of endogenous DX2 and AIMP2, H460 cells were treated with 0, 0.6, 1.2, 2.5 and 5.0 μM pyrimethamine for 12 h. The amounts of DX2 and AIMP2 protein were detected by immunoblotting, and the effect of pyrimethamine on the mRNA transcript levels was monitored via our previously established RT-PCR [9,11]. Pyrimethamine reduced the protein level of DX2, but not AIMP2, (Figure 4a, upper), which is consistent with the result of the nanoluciferase assay described above. However, pyrimethamine did not affect the mRNA transcript level of either DX2 or AIMP2 (Figure 4a, bottom), while our previous hit, BC-DXI01, inhibited DX2 level through decreasing the mRNA transcript level. These results suggest that pyrimethamine selectively decreased the protein level of DX2 with no effect on the mRNA transcript levels. Additionally, we performed an ubiquitination assay [9,11] to confirm pyrimethamine-mediated ubiquitination of DX2 because our previous study reported that DX2 protein was degraded by ubiquitination [24]. As shown in Figure 4b, DX2 was degraded by ubiquitination upon pyrimethamine treatment in a dose-dependent manner. Thus, pyrimethamine appears to induce ubiquitination of DX2 for its degradation.

### 2.5. Inhibitory Effect of Pyrimethamine on Tumorigenesis In Vivo

**Figure 5:**
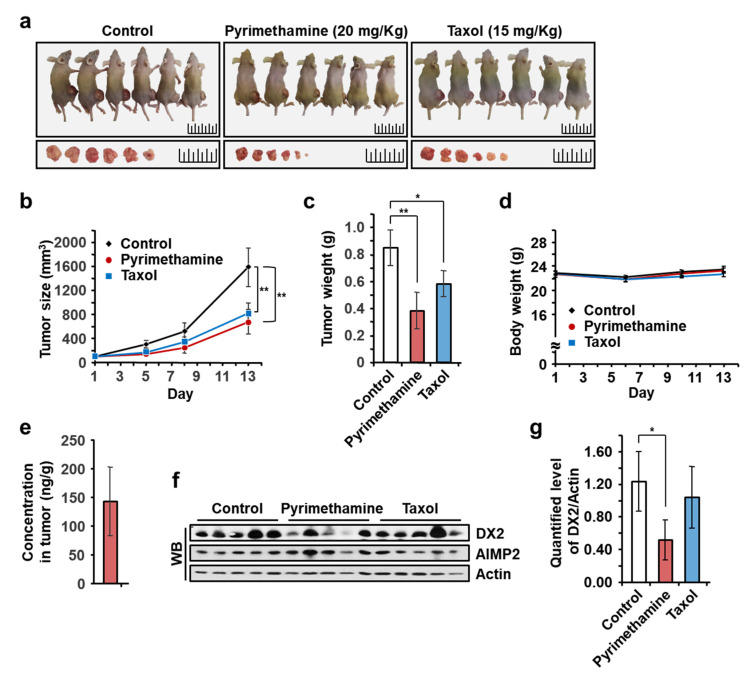
Pyrimethamine suppresses tumor progression in vivo. (**a**–**g**), Xenograft experiment for evaluating the in vivo tumor-suppressive effect of pyrimethamine. Pyrimethamine (20 mg/kg/day) and Taxol (15 mg/kg/day) were intraperitoneally injected, and the tumor sizes (**b**) and body weights (**d**) were measured during the experimental period. After sacrifice, the mice and the excised tumors were photographed (**a**), and the tumors were weighed (**c**). The concentration of pyrimethamine (**e**) and the levels of endogenous DX2 and AIMP2 proteins (**f**,**g**) in the excised tumors were determined by LC-MS/MS and Western blot (WB) analysis, respectively. Average of the quantified values among tumors were presented as a bar graph (**g**). Control means the carrier without chemicals. Student’s two-tailed *t*-test was performed for statistical analysis (* *p* < 0.05, ** *p* < 0.01). The error bars denote the standard deviation (S.D.), and the data are shown as the mean ± S.Ds.

We next examined the in vivo antitumor activities of pyrimethamine in a BALB/cSLC-*nu/nu* mouse xenograft model using H460 cells, which exhibited the most potent growth inhibition among the tested lung cancer cells. When the embedded tumor mass reached a volume of approximately 100 mm^3^, pyrimethamine (20 mg/kg/day, at 1–5 and 8–12 day) and taxol (15 mg/kg/day, at 1, 3, 5, and 8, 10, 12 day) were intraperitoneally injected five and three times a week, respectively, for two weeks. We decided the dosage of pyrimethamine as 20 mg/kg/day, because BC-DXI-01 with a half efficacy of pyrimethamine on cell showed in vivo efficacy at 50 mg/kg/day [11]. Taxol was treated as the published method [25]. Tumor growth in the pyrimethamine- and taxol-treated groups was apparently inhibited compared to that in the control group (Figure 5a). Tumor volumes and weights were reduced by up to 40% following pyrimethamine treatment (Figure 5b,c), while pyrimethamine did not affect the body weights of treated mice (Figure 5d). The concentration of the injected pyrimethamine in tumor tissue was 142 ng/g, high enough to decrease the endogenous DX2 level (Figure 5e). The effects of pyrimethamine on endogenous DX2 or AIMP2 were also analyzed by immunoblotting, and the specific decrease of DX2, not AIMP2, protein level was frequently observed in the pyrimethamine-treated tumor tissues (Figure 5f,g).

## 3. Discussion

Through the screening of a drug repositioning library from KCB, pyrimethamine was identified as a hit chemical for the treatment of lung cancer, selectively inhibiting DX2 over AIMP2 (IC_50_ > 100 µM) with an IC_50_ value of 0.73 µM in A549 cells expressing nanoluciferase-tagged DX2. It also decreased the cell viability of DX2-inducible A549 cells but showed no effect on empty vector-inducible A549 cells, indicating that the DX2 level is important for pyrimethamine-mediated suppression of A549 cell viability. Additionally, cell viability assays on 5 lung cancer cell lines with various expression levels of DX2 were performed. Pyrimethamine suppressed cell growth most potently in H460 cells, which express a high level of DX2 (GI_50_ = 0.01 µM), followed by A549 (GI_50_ = 0.73 µM), HCC-1359 (GI_50_ = 14.3 µM), HCC-366 (GI_50_ = 22.3 µM), and H2087 (GI_50_ > 100 µM) cells. The potency of cell growth inhibition was well correlated with DX2 level in all cell lines, while taxol showed similar potencies in all 6 tested cell lines (GI_50_ = 0.02~0.08 µM). An immunoblot assay of H460 cells treated with pyrimethamine showed decreased level of DX2 in dose dependent manner. While pyrimethamine decreased the protein level of DX2, it did not decrease the DX2 mRNA transcript level, differently from our previous hit, BC-DXI-01 to decrease the DX2 mRNA [11]. Pyrimethamine also showed increased potency than BC-DXI-01 with better pharmacokinetic properties. Pyrimethamine was confirmed to induce ubiquitin-mediated DX2 degradation. Finally, we evaluated in vivo efficacy of pyrimethamine in a mouse xenograft assay using H460 cells. It significantly reduced the tumor size and weight, comparable with the effects of taxol, without affecting body weight. Analysis of tumor tissue showed a considerably high concentration of pyrimethamine with a decreased level of DX2. In this experiments, pyrimethamine showed potent antitumorigenic efficacy against H460 lung cancer cells through decreasing the DX2 protein level.

Currently, pyrimethamine is used for the treatment of parasite diseases including toxoplasmosis, actinomycosis, and isosporiasis, and for the treatment and prevention of *Pneumocystis jirovecii pneumonia* through the competitive inhibition of DHFR, thereby interfering with the regeneration of tetrahydrofolic acid from dihydrofolate [26]. Tetrahydrofolic acid is essential for DNA and RNA synthesis in many species, including protozoa. Antitumor effects of pyrimethamine through targeting DHFR were reported [16,20]. STAT3 has been intensively studied as a therapeutic target for cancer of pyrimethamine including lung cancer [21,23]. There is a report that DHFR reduced STAT3 activity [27], while the exact mechanism of action on antitumor effects of pyrimethamine is not still clear. In this study, we showed that pyrimethamine induced the ubiquitination of DX2, which might provide one of the plausible mechanism on the antitumor efficacy of pyrimethamine against cancer cells with high level of DX2. Pyrimethamine can cause gastrointestinal symptoms such as nausea, vomiting, anorexia, and diarrhea when higher doses are used (www.drugs.com). Because pyrimethamine inhibited lung cancer cells potently, it may be repositioned to treat lung cancer patients with an enough safety margin. Also, it needs further safety study to be used in cancer because the duration of treatment on cancer would be longer compared to infectious diseases. We are continuously trying to validate the efficacy and safety of pyrimethamine as an anti-cancer agent, and elucidate its mode of action.

## 4. Materials and Methods

### 4.1. Cell Culture and Materials

Five lung cancer cell lines, namely, A549, H460, HCC-1359, HCC-366 and H2087, were cultured in RPMI medium supplemented with 10% FBS and 1% penicillin/streptomycin in 5% CO_2_ at 37 °C. WI-26 and 293T cells were cultured in DMEM medium under the same conditions described above. All used cell lines were purchased and authenticated from Korea Cell Line Bank, utilizing Short Tanden Repeat (STR) profiling. The specific antibodies against DX2 and AIMP2 were kindly provided by the biobank of Biocon (Medicinal Bioconvergence Research Center, Yonsei University, Incheon, Korea) [9,21]. Specific antibodies against Actin (#A1978), HA (SC-7392) and Strep (#2-1509-001) were purchased from Sigma (St. Louis, MO, USA), Santa Cruz Biotechnology and IBA, respectively. MG-132 (#474790) and doxycycline (Dox, #D3447) were purchased from Millipore (Burlington, MS, USA) and Sigma (St. Louis, MO, USA), respectively.

### 4.2. Library Screening Based on a Nanoluciferase Assay

A549 cells (4 × 10^4^ cells/mL) expressing nanoluciferase-tagged DX2 via transient transfection were seeded and cultured in 96-well white flat bottom plates (#3903 Corning) for 12 h. Cells were incubated in serum-free medium containing 763 compounds (5 μM) in a drug repositioning library from Korea Chemical bank (KCB) for 4 h, and the luminescence signal was measured following the manufacturer’s protocol (Promega, Madison, WI, USA). The instrument used for measuring the luminescence is GloMax discover (Promega). Thirty chemicals showing greater than 50% inhibition in the primary screen at 5 µM were subjected to secondary screening via a luciferase assay using nanoluciferase-tagged AIMP2 for negative screening, as described above. The IC_50_ values, were calculated using the Prism (GraphPad, San Diego, CA, USA).

### 4.3. Cell Viability Assay

A549, H460, HCC-1359, HCC-366, H2087 (1.5 × 10^4^ cells/mL) and WI-26 cells (4 × 10^4^ cells/mL) were seeded and cultured in 96-well flat bottom plates for 24 h and treated with chemicals in serum-free medium for 72 h. After incubation, 10 μL of MTT solution (5 mg/mL, Sigma) was added and incubated for 1.5 h at 37 °C. After the medium containing MTT solution was discarded, the precipitated formazan crystals were dissolved in 100 μL of DMSO (Duchefa) per well. The absorbance was measured at 560 nm using a microplate reader (Sunrise, TECAN, Männedorf, Switzerland). GI_50_ was calculated by using the Prism (GraphPad).

### 4.4. Assessment of Cell Viability Using DX2- and EV (Empty Vector)-Inducible A549

To assess the DX2-dependent effect of pyrimethamine on cell viability, DX2 was cloned into the EcoRI/NotI sites of pTetOne vector (Promega). pTetOne vector cloned with DX2 or vector only for expressing empty vector was transfected into A549 cells using transfection reagent, lipofectamine2000 (Invitrogen, Carlsbad, CA, USA) following the instruction. A549 cells were subjected to selection by treatment with puromycin (Sigma) every two days for two weeks. EV- and DX2-inducible A549 cells (1 × 10^4^ cells/mL) were cultured in 96-well plates for 24 h, and doxycycline (1 μg/mL) was then added for induction. After induction for 6 days, the isogenic cell lines were incubated in serum-free medium with pyrimethamine (1 µM). Cell viability was assessed with a cell viability assay.

### 4.5. Immunoblotting and RT-PC

Cells were lysed with 50 mM Tris-HCl (pH 7.4) lysis buffer containing 100 mM NaCl, 0.5% Triton X-100, 0.1% SDS, 10% glycerol, 1 mM EDTA, and a protease inhibitor (Calbiochem, San Diego, CA, USA), for 45 min. Supernatants from cell lysates were subjected to SDS-PAGE for protein separation. Proteins of interest were detected by immunoblotting using a corresponding specific antibodies. Total RNA was extracted from H460 cells with an RNeasy Mini Kit (Qiagen, Valencia, CA, USA) and reverse transcriptase polymerase chain reaction (RT-PCR) was conducted with 1 μg of the extracted RNA, dNTPs, random hexamers and Moloney murine leukemia virus (MMLV) transcriptase. To measure the mRNA expression of DX2, AIMP2 and Actin, 2 μL of cDNA was used for PCR with the corresponding specific primers. The specific primer sequences were as follows. Simultaneous detection of both DX2 and AIMP2: ATGCCGATGTACCAGGTAAAG and CTTAAGGAGCTTGAGGGCCGT; Actin: CCTTCCTGGGCATGGAGTCCT and GGAGCAATGATCTTGATCTT. Actin was used as a loading control.

### 4.6. Ubiquitination Assay

The detailed procedures were following our previous report [12]. 293T cells expressing HA-ubiquitin and Strep-DX2 were treated with various doses of pyrimethamine and MG-132 (50 μM) for 12 h. Cells were lysed with 50 mM Tris-HCl (pH 7.4) lysis buffer containing 100 mM NaCl, 0.5% Triton X-100, 0.1% SDS, 10% glycerol, 1 mM EDTA, and a protease inhibitor (Calbiochem). Total cell lysates were precipitated with a Strep-Tactin column, and the eluates were separated by SDS-PAGE. The level of ubiquitinated DX2 was determined by immunoblotting using a specific antibody against HA.

### 4.7. Animals

Seven-week-old female BALB/cSLC-*nu/nu* mice (Central Lab. Animal Inc., Seoul, Korea), were used in this study. Mice were bred in specific pathogen free (SPF) condition. Animal experiments were performed in compliance with the guidelines of the University Animal Care and Use Committee at Seoul National University (SNU-150901-2, SNU-181205-9).

### 4.8. In Vivo Mouse Xenograft

H460 cells (1 × 10^7^) were subcutaneously injected into the backs of 7-week-old female BALB/cSLC-*nu/nu* mice (Central Lab. Animal Inc., Korea) (n = 5 mice/group). Pyrimethamine (20 mg/kg) and Taxol (15 mg/kg) were intraperitoneally administered five and three times a week, respectively, for 2 weeks. Pyrimethamine and Taxol were treated at 0, 24, 48, 72, 96, 168, 192, 216, 240, and 264 h (10 times) and at 0, 48, 96, 168, 216, and 264 h (6 times), respectively. The used carrier for control was DTPP (DMSO: Tween 80: PEG400: PBS = 20:1:30:49). The embedded tumor sizes and body weight were measured four times during the experimental period. After 13 days, all mice were sacrificed, and the mice with embedded tumors, along with the excised tumors, were photographed. After the harvested tumors were weighed, the endogenous protein levels of DX2 and AIMP2 were determined by immunoblotting. The tumors were lysed in PBS containing 1% Triton X-100, 0.1% SDS and a protease inhibitor (Calbiochem), and supernatants from the extracts were subjected to SDS-PAGE and immunoblotting using specific antibodies against DX2 and AIMP2. To measure the concentration of pyrimethamine in the excised tumors, the harvested tumors were homogenized in three volumes of PBS buffer and mixed with nine volumes of cold acetonitrile containing the internal standard (disopyramide). After centrifugation at 15,000 rpm and 4 °C for 10 min, 100 μL of the supernatants was subjected to LC-MS/MS analysis [28]. Mass spectrometry (Agilent 6460) with HPLC (Agilent 1261) was used for mass analysis. Standard samples were prepared via the same procedure described above.

### 4.9. Statistics

Statistical tests were performed with Prism (GraphPad). A value of *p* < 0.05 was considered statistically significant. All error bars means standard deviation (S.D.). Statistical parameters are presented in the figure legends.

## 5. Conclusions

In this study, pyrimethamine displayed selective inhibitory activity against level of DX2 over AIMP2 in A549 cells expressing nanoluciferase-tagged DX2, and most potently suppressed the growth of H460 cells, which express high levels of DX2. Pyrimethamine also decreased the DX2 level dose-dependently but did not affect the AIMP2 level in an immunoblot assay using H460 cells. Further experiments confirmed that pyrimethamine resulted in ubiquitination-mediated DX2 degradation. In an in vivo mouse xenograft assay using H460 cells, pyrimethamine significantly reduced the tumor size and weight, comparable with the effects of taxol, without affecting body weight. In conclusion, pyrimethamine showed potent antitumorigenic efficacy against H460 lung cancer cells through decreasing the DX2 protein level.

## Figures and Tables

**Table 1:** Inhibition of lung cancer growth by pyrimethamine.

Cell Line	DX2 Level	GI_50_ (µM) ^1^	
		Pyrimethamine	Taxol
H460	high	0.01	0.02
A549 HCC-1359 HCC-366	moderate	0.8 14.3 22.3	0.03 0.08 0.05
WI-26 H2087	low	>100 >100	0.04 0.03

^1^ GI_50_ values are average of three independent experiments.

## References

[1] Rajendran V., Kalita P., Shukla H., Kumar A., Tripathi T. (2018). Aminoacyl-tRNA synthetases: Structure, function, and drug discovery. Int. J. Biol. Macromol..

[2] Park S.G., Ewalt K.L., Kim S. (2005). Functional expansion of aminoacyl-tRNA synthetases and their interacting factors: New perspectives on housekeepers. Trends Biochem. Sci..

[3] Kim S., You S., Hwang D. (2011). Aminoacyl-tRNA synthetases and tumorigenesis: More than housekeeping. Nat. Rev. Cancer.

[4] Cho H.Y., Maeng S.J., Cho H.J., Choi Y.S., Chung J.M., Lee S., Kim H.K., Kim J.H., Eom C.Y., Kim Y.G. (2015). Assembly of multi-tRNA synthetase complex via heterotetrameric glutathione tranferase-homology domains. J. Biol. Chem..

[5] Kim M.J., Park B.-J., Kang Y.-S., Kim H.J., Park J.-H., Kang J.W., Lee S.W., Han J.M., Lee H.-W., Kim S. (2003). Downregulation of FUSE-binding protein and c-myc by tRNA synthetase cofactor p38 is required for lung cell differentiation. Nat. Genet..

[6] Choi J.W., Kim D.G., Park M.C., Um J.Y., Han J.M., Park S.G., Choi E.C., Kim S. (2009). AIMP2 promotes TNF-dependent apoptosis via ubiquitin-mediated degradation of TRAF2. J. Cell Sci..

[7] Han J.M., Park B.-J., Park S.G., Oh Y.S., Choi S.J., Lee S.W., Hwang S.-K., Chang S.-H., Cho M.-H., Kim S. (2008). AMIP2/p38, the scaffold for the multi-tRNA synthetase complex, responds to genetoxic stresses via p53. Proc. Natl. Acad. Sci. USA.

[8] Choi J.W., Um J.Y., Kundu J.K., Surh Y.J., Kim S.K. (2009). Multidirectional tumor-suppressive activity of AIMP2/p38 and the enhanced susceptibility of AIMP2 heterozygous mice to carcinogenesis. Carcinogenesis.

[9] Choi J.W., Kim D.G., Lee A.E., Kim H.R., Lee J.Y., Kwon N.H., Shin Y.K., Hwang S.K., Chang S.H., Cho M.H. (2011). Cancer-associated splicing variant of tumor suppressor AIMP2/p38: Pathological implication in tumorigenesis. PLoS Genet..

[10] Choi J.W., Lee J.W., Kim J.K., Jeon H.K., Choi J.J., Kim D.G., Kim B.G., Nam D.H., Kim H.J., Yun S.H. (2012). Splicing variant of AIMP2 as an effective target against chemoresistant ovarian cancer. J. Mol. Cell. Biol..

[11] Lee H.S., Kim D.G., Oh Y.S., Kwon N.H., Lee J.Y., Kim D., Park S.-H., Song J.-H., Lee S., Han J.M. (2013). Chemical suppression of an oncogenic splicing variant of AIMP2 induces tumor regression. Biochem. J..

[12] https://www.chembank.org.

[13] Montoya J.G., Lisenfeld O. (2004). Tpxoplasmosis. Lancet.

[14] Hopper A.T., Brockman A., Wise A., Gould J., Barks J., Radke J.B., Sibley L.D., Zou Y., Thomas S. (2019). Discovery of selective *Toxoplasma gondii* diyydrofolate reductase inhibitors for treatment of toxoplasmosis. J. Med. Chem..

[15] Hujisduijnen R.H., Guy R.K., Chibale K., Haynes R.K., Peitz I., Kelter G., Phillips M.A., Vennerstorm J.L., Yuthavong Y. (2013). Anticancer properties of distinct antimalarial drug classes. PLoS ONE.

[16] Chen M., Osman I., Orlow S.J. (2009). Amtifolate activity of pyrimethamine enhances temozomide-induced cytotoxicity in melanoma cells. Mol. Cancer Res..

[17] Giammarioli A.M., Maselli A., Casagrande A., Gambardella L., Gallina A., Spada M., Giovannetti A., Proietti E., Walter M. (2008). Pyrimethamine induces apoptosis of melanoma cells via a caspase and cathepsin double edged mechanism. Cancer Res..

[18] Dai C., Zhang B., Liu X., Guo K., Ma S., Cai F., Yang Y., Yao Y., Feng M., Bao X. (2013). Pyrimethamine sensitizes pituitary adenomas cells to temozolamide through cathepsn B-dependent and caspase dependent pathways. Int. J. Cancer.

[19] Lin M.-X., Lin S.-H., Lin C.-C., Yang C.-C., Yuan S.-Y. (2018). In vitro and in vivo antitumor effects of pyrimethamine on non-small cell lung cancers. Anticancer Res..

[20] Liu H., Qin Y., Zhai D., Zhang Q., Gu J., Tang Y., Yang J., Li K., Yang L., Chen S. (2019). Antimalarial drug pyrimethamine plays a dual role in antitumor proliferation and metastasis through targeting DHFR and TP. Mol. Cancer Res..

[21] Khan M.W., Saadalla A., Ewida A.H., Al-Katranji K., Al-Saoudi G., Giaccone Z.T., Gounari F., Zhang M., Frank D.A., Khazai K. (2018). The STAT3 inhibitor pyrimethamine displays anti-cancer and immune stimulatory effects in murine models of breast cancer. Cancer Immunol. Immunother..

[22] Liu Y., Zhou H., Wang H. (2019). Pyrimethamine Exerts significant antitumor effects on human ovarian cancer cells both in vitro and *in vivo*. Anticancer Drugs.

[23] Qin J.-J., Yan L., Zhang J., Zhang W.-D. (2019). STAT3 as a potential therapeutic target in triple negative breast cancer: A systematic review. J. Exp. Clin. Res..

[24] Lim S., Cho H.Y., Kim D.G., Roh Y., Son S., Mushtaq A.U., Kim M., Bhattarai D., Sivaraman A., Lee Y. (2020). Targeting the interaction of AIMP2-DX2 with HSP70 suppresses cancer development. Nat. Chem. Biol..

[25] Huang S.-T., Wang Y.-P., Chen Y.-H., Lin C.-T., Li W.-S., Wu H.-C. (2018). Liposomal paclitaxel induces fewer hematopoietic and cardiovascular complications than bioequivalent doses of Taxol. Int. J. Oncol..

[26] Hamilton R. (2015). Tarascon Pocket Phramacopoeia, Deluxe Lab-Coat Edition.

[27] Heppler L.N., Walker S.R., Attarha S., Page B.D., Frank D.A. Pyrimethamine inhibits STAT3 transcriptional activity via dihydrofolate reductase. Proceedings of the AACR Annual Meeting.

[28] Song J.S., Rho H.J., Park J.S., Kim M.S., Lee B.H., Seo J.W., Jeon D.J., Cheon H.G., Ahn S.H., Kwon K. (2011). Preclinical pharmacokinetics of PDE-310, a novel PDE4 inhibitor. Drug Metab. Pharmacokinet..

